# Meniscus-Derived Matrix Bioscaffolds: Effects of Concentration and Cross-Linking on Meniscus Cellular Responses and Tissue Repair

**DOI:** 10.3390/ijms21010044

**Published:** 2019-12-19

**Authors:** Lucas P. Lyons, Sofia Hidalgo Perea, J. Brice Weinberg, Jocelyn R. Wittstein, Amy L. McNulty

**Affiliations:** 1 Department of Orthopaedic Surgery, Duke University School of Medicine, Durham, NC 27710, USA; lucas.lyons@duke.edu (L.P.L.); sofia.hidalgo.perea@duke.edu (S.H.P.); jocelyn.wittstein@duke.edu (J.R.W.); 2 Department of Biology, Duke University, Durham, NC 27708, USA; 3 Department of Medicine, VA Medical Center, Durham, NC 27705, USA; brice@duke.edu; 4 Department of Medicine, Duke University School of Medicine, Durham, NC 27710, USA; 5 Department of Pathology, Duke University School of Medicine, Durham, NC 27710, USA

**Keywords:** tissue engineering, regeneration, joint, knee, fibrochondrocyte, biomechanical, cartilage, proteoglycan

## Abstract

Meniscal injuries, particularly in the avascular zone, have a low propensity for healing and are associated with the development of osteoarthritis. Current meniscal repair techniques are limited to specific tear types and have significant risk for failure. In previous work, we demonstrated the ability of meniscus-derived matrix (MDM) scaffolds to augment the integration and repair of an in vitro meniscus defect. The objective of this study was to determine the effects of percent composition and dehydrothermal (DHT) or genipin cross-linking of MDM bioscaffolds on primary meniscus cellular responses and integrative meniscus repair. In all scaffolds, the porous microenvironment allowed for exogenous cell infiltration and proliferation, as well as endogenous meniscus cell migration. The genipin cross-linked scaffolds promoted extracellular matrix (ECM) deposition and/or retention. The shear strength of integrative meniscus repair was improved with increasing percentages of MDM and genipin cross-linking. Overall, the 16% genipin cross-linked scaffolds were most effective at enhancing integrative meniscus repair. The ability of the genipin cross-linked scaffolds to attract endogenous meniscus cells, promote glycosaminoglycan and collagen deposition, and enhance integrative meniscus repair reveals that these MDM scaffolds are promising tools to augment meniscus healing.

## 1. Introduction

The menisci are semi-lunar fibrocartilaginous structures that are essential for the proper biomechanical function of the knee joint [1,2,3,4,5]. They are composed of a highly organized, dense network of collagen fibrils and proteoglycans that work together to promote load transmission across the articular cartilage lining the femoral condyles and tibial plateau [4,6,7,8]. Meniscal injuries are common and occur frequently due to sports-related trauma and/or degenerative changes in the joint. In addition to the initial pain and disability from meniscus injury, approximately 50% of patients with a meniscus tear will develop osteoarthritis within 10–20 years following injury [1,5,9,10]. Total or partial meniscectomy is frequently performed to reduce pain and mechanical symptoms but has been strongly associated with the subsequent development of osteoarthritis [11,12,13]. Despite this, partial meniscectomy remains one of the most commonly performed orthopaedic procedures [1,14]. Meniscus repair procedures are frequently performed in the peripheral, vascularized region of the tissue. However, the inner, avascular region of the tissue has a low healing capacity and thus is less amenable to repair [5,15,16]. Therefore, strategies are needed to preserve meniscal tissue, augment current meniscal repair techniques, and expand indications for meniscus repair.

Tissue-derived scaffolds provide an attractive approach for enhancing meniscus repair by providing a scaffold composed of native extracellular matrix (ECM) components that may promote integrative repair with the endogenous injured meniscus tissue [4,5,17,18,19]. Tissue-derived scaffolds contain tissue-specific growth factors that may facilitate cell infiltration and proliferation, as well as matrix synthesis with minimal need for exogenous growth factors [17,19,20,21,22,23]. Our earlier work demonstrated the ability of meniscus-derived matrix (MDM) scaffolds to promote endogenous meniscus cell migration and enhance the integrative repair of an in vitro meniscus defect [19]. In particular, we determined that 8% MDM scaffolds enhanced meniscus repair as compared to 4% MDM scaffolds, suggesting that higher percentages of MDM may further improve meniscus repair. 

In order to enhance the integrity and mechanical properties of biomaterial scaffolds, a variety of cross-linking strategies have been evaluated, including both physical and chemical cross-linking methods [19,23,24,25,26,27,28,29]. Physical cross-linking methods include dehydrothermal (DHT) treatment [18,19,23,25,29] and exposure to ultraviolet (UV) light [25,26,29], while chemical cross-linking can be achieved with glutaraldehyde [27,28] and genipin [24,30]. DHT cross-linking is known to mediate uniaxial tensile strength of collagen scaffolds [25] and to increase cellular proliferation and proteoglycan production when compared to UV cross-linking of cartilage-derived matrix (CDM) scaffolds [23]. Genipin and glutaraldehyde cross-linking of porcine pericardium results in comparable mechanical strength [31], but genipin is considerably less cytotoxic than glutaraldehyde [24,31,32]. Therefore, the objective of this study was to determine the effects of percent composition and DHT or genipin cross-linking of MDM bioscaffolds on primary meniscus cellular responses and integrative meniscus repair using an in vitro meniscus repair model system. 

## 2. Results

### 2.1. Scanning Electron Microscopy of MDM Scaffolds

**Figure 1:**
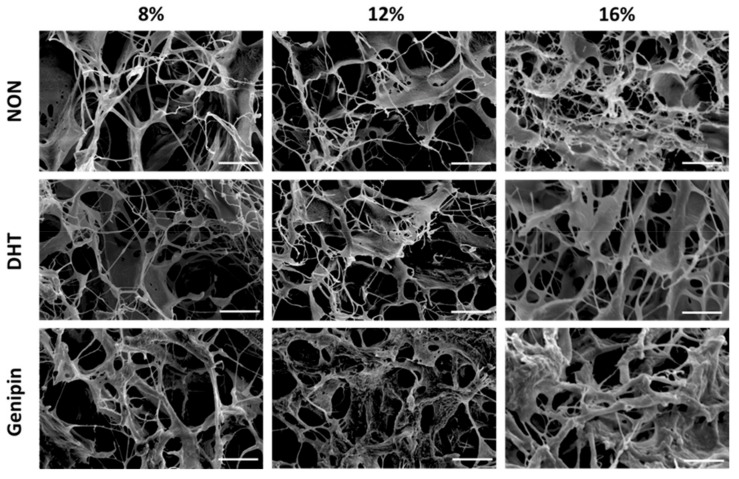
Scanning electron microscopy images showing the architecture of the non-cross-linked (NON), dehydrothermal (DHT) cross-linked, and genipin cross-linked scaffolds composed of different percentages of meniscus-derived matrix (MDM). Scale bar is 10 µm.

Menisci were pulverized to generate MDM powder that was reconstituted to generate 8%, 12%, and 16% MDM scaffolds. These scaffolds were then either left non-cross-linked, or were DHT or genipin cross-linked. We used scanning electron microscopy to visualize the architecture of the scaffolds. There was an apparent increase in ECM components with increasing MDM percentage (Figure 1). As well, genipin cross-linking resulted in thicker aggregates of ECM components. Overall, the MDM scaffolds maintained a porous structure, which could facilitate cell migration into the scaffolds. 

### 2.2. Biochemical Response of Primary Meniscus Cells Seeded on MDM Scaffolds

**Figure 2:**
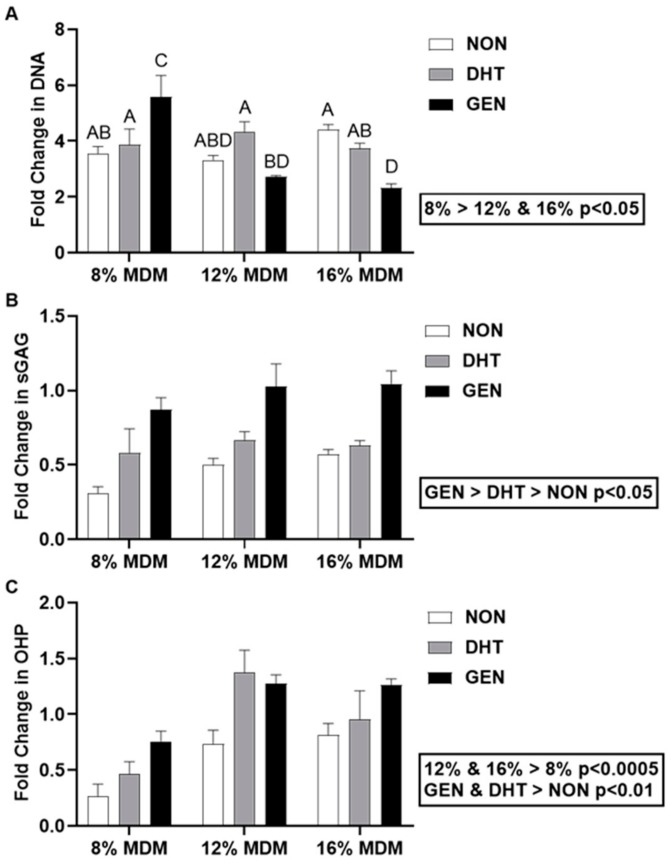
Biochemical properties of the non-cross-linked (NON), dehydrothermal (DHT) cross-linked, and genipin (GEN) cross-linked MDM scaffolds seeded with primary meniscus cells. Fold change (as compared to day 0 meniscus cell-seeded scaffolds) in (**A**) DNA content, (**B**) sulfated glycosaminoglycan (sGAG) content, and (**C**) collagen (OHP) content over 14 days. Data are expressed as the mean + SEM. All groups not sharing the same letter have *p*-values < 0.05.

Meniscus cell-seeded MDM scaffolds were harvested at days 0 and 14, and biochemical analyses were performed. Fold changes in DNA, sulfated glycosaminoglycan (sGAG), and collagen content were determined by normalizing the day 14 biochemical properties to corresponding day 0 meniscus cell-seeded scaffold properties. The 8% MDM scaffolds had a significantly higher change in DNA content as compared to both the 12% and 16% MDM scaffolds (Figure 2A, *p* < 0.05). The 8% genipin cross-linked scaffolds showed the greatest increase in DNA content (*p* < 0.05). In contrast, the 16% genipin cross-linked scaffolds showed the lowest change in DNA content (*p* < 0.05), except when compared to 12% non-cross-linked and 12% genipin cross-linked scaffolds. There was no significant effect of cross-linking on fold change in DNA content. Importantly, all of the MDM scaffolds increased DNA content from day 0 (day 0 = 1). The type of cross-linking significantly affected the sGAG content of meniscus-seeded MDM scaffolds. Genipin cross-linked scaffolds had the greatest change in sGAG content (Figure 2B, *p* < 0.0005), while non-cross-linked scaffolds had the least change (*p* < 0.05). At day 0, the 12% and 16% scaffolds had significantly higher sGAG content than the 8% scaffolds. However, there was no effect of MDM percentage on fold change in sGAG content. The 12% and 16% MDM scaffolds had a greater increase in collagen content than the 8% MDM scaffolds (Figure 2C, *p* < 0.0005). On the other hand, non-cross-linked scaffolds had the lowest change in collagen content as compared to both the DHT and genipin cross-linked scaffolds (*p* < 0.01). 

### 2.3. Meniscus Repair Model System

We used our in vitro meniscus repair model system [18,19,33,34,35,36,37,38,39] to study the integrative repair of acellular MDM scaffolds with meniscus tissue [19]. After 14 days in culture, constructs were analyzed by fluorescence imaging, biochemical analyses, mechanical testing, and histology. 

#### 2.3.1. Fluorescence Imaging

**Figure 3:**
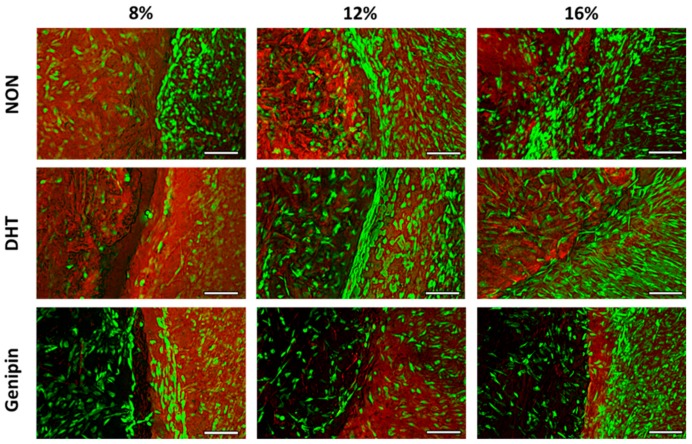
Fluorescent images of the interface between the non-cross-linked (NON), dehydrothermal (DHT) cross-linked, or genipin cross-linked MDM scaffolds and meniscus tissue in the meniscus repair model system after 14 days in culture. Live meniscus cells (green) and ECM proteins (red) are stained. The MDM scaffolds are on the left of each image and the meniscus tissue is on the right. Scale bar is 100 µm.

We performed fluorescence imaging to visualize live cells and ECM at the interface between the MDM scaffolds and the meniscus tissue (Figure 3). Native meniscus cells predominantly filled the interface between the meniscus tissue and the MDM scaffolds. However, in the 8% scaffolds, fewer cells were noted in the interface. In all of the MDM scaffolds, native meniscus cells from the outer tissue ring migrated into the MDM scaffolds. Fewer meniscus cells appeared to have infiltrated the 12% and 16% genipin cross-linked scaffolds as compared to the corresponding non-cross-linked and DHT cross-linked scaffolds. 

#### 2.3.2. Biochemical Analyses of MDM Scaffolds

**Figure 4:**
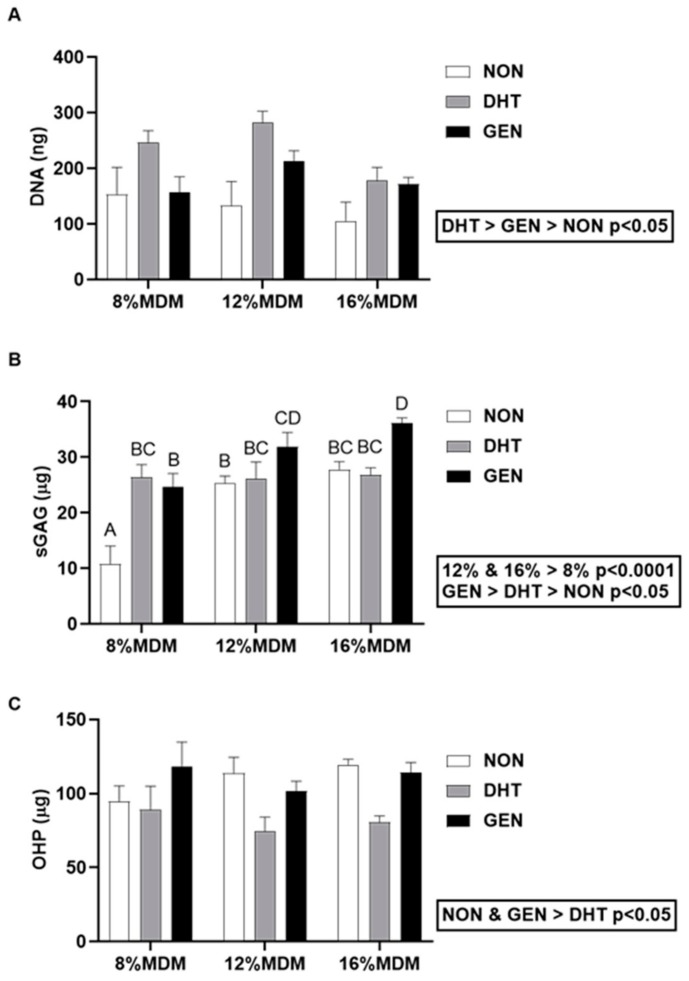
Biochemical composition of the non-cross-linked (NON), dehydrothermal (DHT) cross-linked, and genipin (GEN) cross-linked MDM scaffolds following culture in the meniscus repair model system. (**A**) DNA content, (**B**) sulfated glycosaminoglycan (sGAG) content, and (**C**) collagen (OHP) content after 14 days in culture. Data are expressed as the mean + SEM.

We digested the MDM scaffolds from the in vitro meniscus repair model system after 14 days in culture, and then determined their DNA, sGAG, and collagen contents. There was a significant effect of cross-linking on the DNA content (Figure 4A). The DNA content was highest in the DHT cross-linked scaffolds and lowest in the non-cross-linked scaffolds (*p* < 0.05). However, there was no effect of percent MDM on the DNA content of the scaffolds following culture with meniscus tissue. The 12% and 16% MDM scaffolds had significantly higher sGAG content than the 8% MDM scaffolds (Figure 4B, *p* < 0.0001). In addition, the genipin cross-linked scaffolds had the highest sGAG content (*p* < 0.05), while the non-cross-linked scaffolds had the lowest (*p* < 0.05). Cross-linking and percent MDM had an interactive effect, such that the 8% non-cross-linked scaffolds had the lowest sGAG content (*p* < 0.0001) and the 16% genipin cross-linked scaffolds had the highest sGAG content (*p* < 0.01). However, there was no significant difference in sGAG content between 16% genipin cross-linked and 12% genipin cross-linked scaffolds. The collagen content, measured by hydroxyproline (OHP), was significantly higher in the non-cross-linked and genipin cross-linked scaffolds, as compared to the DHT cross-linked scaffolds (Figure 4C, *p* < 0.005). However, there was no effect of percent MDM on the collagen content of the scaffolds following culture with meniscus tissue. 

#### 2.3.3. Integrative Shear Strength of Repair

**Figure 5:**
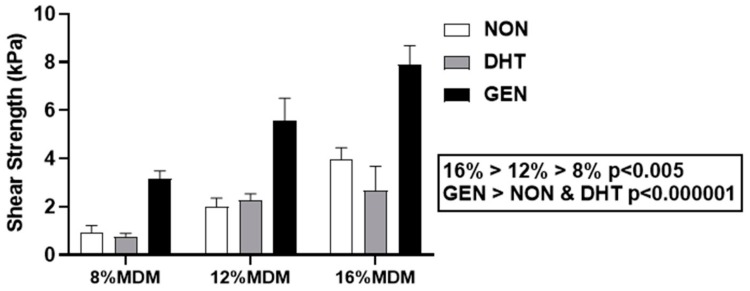
Integrative shear strength of repair of the non-cross-linked (NON), dehydrothermal (DHT) cross-linked, and genipin (GEN) cross-linked MDM scaffolds with meniscus tissue in the meniscus repair model system. Data is expressed as the mean + SEM.

Push-out testing was used to quantify the integrative shear strength of repair [18,19,33,34,35,36,37,38,39] between the MDM scaffolds and meniscus tissue after 14 days in culture. The integrative shear strength of repair increased with the concentration of MDM in the scaffolds, such that 16% > 12% > 8% (Figure 5, *p* < 0.005). There was also a significant effect of cross-linking, revealing that the genipin cross-linked scaffolds had the highest shear strength of repair compared to both the non-cross-linked and DHT cross-linked scaffolds (*p* < 0.000001). The shear strength of repair for the native meniscus tissue control (meniscus tissue inner core and outer ring) was 5.3 ± 1.5 kPa (mean ± SEM). 

#### 2.3.4. Histological Assessment

**Figure 6:**
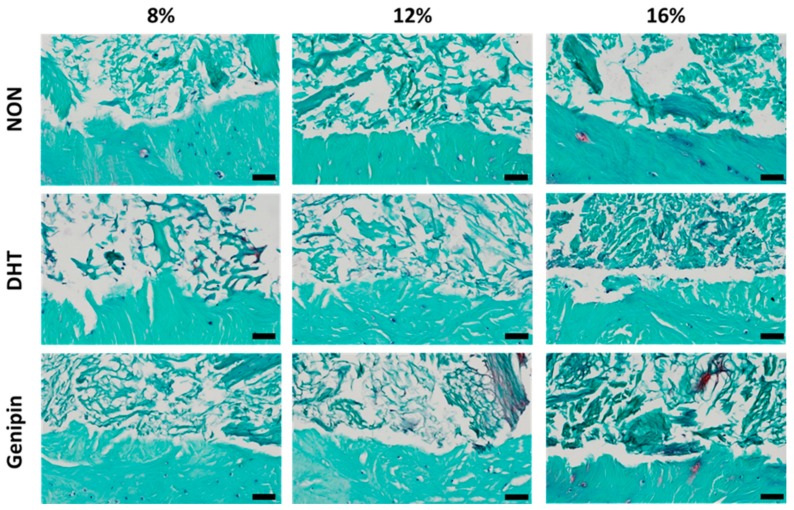
Histological images of the interface between the non-cross-linked (NON), dehydrothermal (DHT) cross-linked, and genipin cross-linked MDM scaffolds and meniscus tissue in the meniscus repair model system after 14 days in culture. Each image is a sagittal cross-section with the meniscus tissue at the bottom of the frame and the MDM scaffold at the top. Tissues were stained with hematoxylin (black), fast green (green), and safranin-O (red) to visualize nuclei, collagen, and proteoglycans respectively. Scale bar is 50 µm.

After 14 days in culture, we stained histological sections with hematoxylin, fast green, and safranin-O, to visualize nuclei, collagen, and proteoglycans, respectively. Staining revealed that all MDM scaffolds had integrated with the surrounding meniscus tissue (Figure 6). In addition, endogenous meniscus cells migrated into all scaffold groups, and the repair tissue localized to the interface was predominately composed of collagen. There was an increase in the density of the ECM components in the higher concentration MDM scaffolds. Furthermore, there was greater proteoglycan staining in the cross-linked scaffolds, as compared to the non-cross-linked scaffolds. 

## 3. Discussion

Our current work investigated the effects of MDM concentration and various methods of cross-linking on both exogenous meniscus cellular responses and on integrative meniscus repair, mediated by endogenous meniscus cells using an in vitro repair model system. Meniscus cells were able to infiltrate and proliferate on all of the MDM scaffolds evaluated in this study. Genipin cross-linked scaffolds promoted ECM production and/or retention. Furthermore, the shear strength of repair was improved by higher percentages of MDM and genipin cross-linking. These findings suggest that the 16% genipin cross-linked MDM scaffolds will likely be a valuable tool to enhance healing with native meniscus tissue to promote tissue repair.

All of the MDM scaffolds evaluated in this study were able to support the infiltration of both seeded meniscus cells and meniscus cell migration from the surrounding meniscus tissue. Furthermore, all of the MDM scaffolds promoted cellular proliferation on the scaffolds, as evidenced by the greater-than-one-fold change in the DNA content of the scaffolds seeded with exogenous meniscus cells. In the meniscus cell seeding experiments, the 8% MDM scaffolds had the highest cellular proliferation. However, there was no effect of MDM concentration on the DNA content of the scaffolds in the meniscus tissue repair model, suggesting that cellular migration and proliferation into the scaffolds from the native meniscus tissue is not influenced by the density of the ECM components in the MDM scaffolds. 

In addition to providing a porous bioscaffold that enables meniscus cell migration, proliferation, and fibrochondrogenic inductive properties, it is important for reparative scaffolds to aid in the restoration of the highly complex organization of the meniscus ECM components. Previous studies have shown that the meniscus ECM micro- and macro-structural organization is essential for the mechanical function of the tissue [40]. The MDM processing procedures used in this study provide the ability to tailor the structure of scaffolds via alterations in the percent composition of MDM, cross-linking strategies, and/or freezing rates [41], to promote alignment of collagen and other ECM components. In addition, the compressive and tensile loading [42] of MDM scaffolds in meniscus defects may further promote the formation of native meniscus ECM hierarchical structure.

Scaffold cross-linking significantly influenced cellular behavior on the MDM scaffolds. Genipin is a natural cross-linking agent derived from geniposide, which is found in the fruit of *Gardenia jasminoides* [43]. It acts by bridging amino acid side chains of adjacent lysine or hydroxylysine residues of neighboring polypeptide chains [24,43,44]. Therefore, genipin is incorporated into the cross-linked fibers. Prior studies have shown that high concentrations of genipin can prevent cellular attachment to scaffolds and reduce cell viability [24,45]. In particular, cross-linking using 0.1–2% genipin reduces the cell metabolic activity of rabbit bone marrow-derived cells but not adipose tissue-derived stem cells (ASCs) on collagen-chitosan scaffolds, as compared to monolayer cells [45]. This may be attributable to the chitosan scaffolds, which can inhibit cellular proliferation [45]. On the other hand, other studies using genipin as a cross-linking method for collagen–chitosan scaffolds have shown no adverse effects on the metabolic activity of chondrocytes or human mesenchymal stem cell (hMSC)-seeded scaffolds [30,45,46]. Furthermore, Cheng and colleagues have shown that cross-linking of CDM by 0.5% genipin reduces ASC viability and proliferation over 28 days in culture [24]. However, there was no effect of cross-linking CDM with 0.05% genipin on ASC viability and cell proliferation. In our study, 0.05% genipin did not reduce cellular attachment at day 0 during meniscus cell seeding. In addition, there was no significant effect of cross-linking on fold change in DNA content of exogenously seeded MDM scaffolds, suggesting that genipin cross-linking did not reduce or inhibit cellular proliferation. Although the 16% genipin cross-linked scaffolds demonstrated the lowest exogenous meniscus cell proliferation, the fold change in DNA content was similar to that found in 12% non-cross-linked and 12% genipin cross-linked scaffolds. Conversely, the 8% genipin cross-linked scaffolds exhibited the highest fold change in DNA content. Together, these results suggest that the reduced proliferation on the genipin cross-linked scaffolds is likely not due to cytotoxicity, but the result of the physical alteration of the MDM scaffolds, particularly at higher concentrations of MDM [24,30,46,47]. 

Similar to the exogenous meniscus cell seeding data, the fluorescent images of the meniscus repair model explants showed fewer meniscus cells in the 12% and 16% genipin cross-linked scaffolds. However, the DNA content of the non-cross-linked scaffolds in the meniscus repair model was significantly lower than the DNA content in the genipin cross-linked scaffolds. This is likely attributable to the greater retention of residual DNA in the higher percentage MDM scaffolds that were genipin cross-linked. The 12% and 16% genipin cross-linked MDM scaffolds retained approximately three-fold more DNA than all other scaffolds, which averaged 20 ng DNA per mg dry weight after scaffold processing. The general recommendation for decellularization is <50 ng DNA per mg dry weight, in order to reduce the risk of immunoreactivity [48]. Prior work has shown that there are no differences in macrophage reactivity in response to implanted collagen scaffolds alone or scaffolds supplemented with 50 ng DNA or equivalent cellular quantities of mitochondria or cell membrane materials [49]. Nonetheless, the average DNA content of our scaffolds was 90% less than that of native meniscus tissue, which is comparable to other protocols using chemical and enzymatic decellularization techniques [50,51,52]. However, these decellularization methods also substantially reduced the proteoglycan content of the scaffolds [51,53,54]. Our MDM scaffolds retained up to 50% of native meniscus tissue sGAG content, even after 14 days in culture, indicating that our MDM scaffold preparation procedure is superior at maintaining meniscus-derived proteoglycans. 

Both physical and chemical cross-linking of the MDM scaffolds were able to enhance retention and/or promote production of sGAGs in the exogenous meniscus cell-seeded scaffolds and scaffolds cultured in the meniscus repair model system. In our experiments, genipin cross-linked scaffolds had the greatest fold change or total sGAG content, while the non-cross-linked scaffolds had the lowest fold change or total sGAG content, revealing the loss of sGAGs from the MDM scaffolds without cross-linking. Consistent with our biochemical results, proteoglycans were detected histologically in the cross-linked scaffolds in the meniscus repair model experiments. Prior work has shown that proteoglycans are leached from meniscus tissue explants during culture [38,55]. However, the genipin cross-linked scaffolds seeded with exogenous meniscus cells maintained initial concentrations of sGAGs throughout culture (fold change ≥1). While other studies have reported a loss of sGAG content during the genipin cross-linking process [24], this study found no differences in the initial sGAG content at day 0 due to cross-linking. In addition to the positive effects of genipin cross-linking on sGAG content, the higher percentage MDM scaffolds (12% and 16%) also exhibited a higher sGAG content than the 8% MDM scaffolds cultured in the meniscus repair model. Specifically, the 16% genipin cross-linked scaffolds exhibited the highest sGAG content, but this group is not significantly different from the 12% genipin cross-linked scaffolds. 

Overall collagen content was improved by the genipin cross-linking of the MDM scaffolds. For the exogenous meniscus cell seeded scaffolds, both genipin and DHT cross-linking resulted in the highest fold change in collagen content. The exogenous meniscus cells on the genipin cross-linked scaffolds maintained initial concentrations of collagen throughout culture (fold change ≥1). In addition, the higher concentrations of MDM promoted an increase in collagen content during culture. However, for scaffolds in the meniscus repair model system, the non-cross-linked and genipin cross-linked scaffolds had the highest collagen content. Prior work with genipin cross-linked CDM scaffolds that were seeded with ASCs revealed the expression of type II collagen and aggrecan with higher expression levels at day 28 compared to earlier time points [24]. Specifically, the 0.05% genipin cross-linked CDM scaffolds resulted in a 1700-fold increase in *COL2A1* transcript levels at day 28 when compared to the non-cross-linked CDM scaffolds [24]. These findings suggest that longer culture times may be necessary to further enhance collagen and proteoglycan production and deposition in the genipin cross-linked scaffolds. In addition, future studies could include radiolabeling to delineate newly synthesized proteoglycans and collagens from ECM components retained in the MDM scaffolds.

The shear strength of integrative meniscus repair was improved with each increasing percentage of MDM, such that 16% MDM resulted in the highest shear strength of repair. This finding is consistent with our previous work, showing that 8% MDM scaffolds enhanced meniscus repair over 4% MDM scaffolds [19]. Also, higher concentrations of CDM have shown an increase in the compressive modulus of CDM scaffolds [41]. However, this relationship between increased repair strength and increased MDM concentration likely would not continue at much higher concentrations. Higher percentage scaffolds have an increased density of ECM components that could negatively influence scaffold porosity and thus cellular infiltration and migration, and likely lead to decreased integrative repair capabilities [56]. This challenge has been observed in studies using whole decellularized menisci for allograft bioscaffolds [57,58]. Whole meniscus allograft bioscaffolds show promising results in both biocompatibility and biomechanical properties when compared to native meniscus. However, they do not facilitate cellular infiltration [59], a process that is likely necessary for long-term regeneration and repair. 

DHT cross-linked scaffolds in the meniscus repair model system have the highest DNA content, but have reduced sGAG and collagen content as compared to the genipin cross-linked scaffolds. It is likely that the reduced ECM content of these scaffolds resulted in the decreased shear strength of meniscus repair. These findings are consistent with our previous work [18,19], demonstrating no differences in the shear strength of repair between 4% non-cross-linked and 4% DHT cross-linked MDM scaffolds or between 8% non-cross-linked and 8% DHT cross-linked MDM scaffolds [19]. More recently, in a novel meniscus defect model filled with MDM scaffolds seeded with hMSCs, we found an increase in the shear strength of repair when the defect was filled with DHT cross-linked MDM as compared to MDM slurry [18]. However, no significant differences were observed between the defects filled with DHT cross-linked MDM scaffolds and non-cross-linked MDM scaffolds [18]. The high heat and long incubation necessary for DHT cross-linking can result in denaturation of tissue-derived proteins, which may negatively influence cell-matrix interactions and mechanical integrity of DHT cross-linked scaffolds [23,25,60].

In this study, we demonstrated the utility of MDM bioscaffolds to enhance meniscus healing. Genipin cross-linked MDM scaffolds were able to support the proliferation and migration of native meniscus cells, promote sGAG and collagen deposition, and enhance integrative meniscus healing through the formation of a collagen rich repair interface. In particular, the shear strength of integrative repair of the 16% genipin cross-linked scaffolds with meniscus tissue was 150% higher than meniscus tissue healing to itself. These scaffolds, which are composed of naturally occurring meniscus-matrix components, are promising tools to augment meniscus repair procedures to enhance tissue healing. These findings lay the groundwork for future preclinical studies to evaluate the MDM scaffolds in an in vivo meniscus repair model, to improve long-term meniscus healing and prevent the development of osteoarthritis.

## 4. Materials and Methods 

### 4.1. Generation of MDM Scaffolds 

MDM was generated as previously described [18,19]. Briefly, medial menisci were harvested from skeletally mature, female porcine tibiofemoral joints obtained from a local abattoir. Menisci were minced into ≤ 5mm pieces, frozen overnight at −80 °C, and lyophilized (FreeZone 2.5 L, Labconco, Kansas City, MO, USA). Dehydrated meniscal pieces were pulverized using a 6770 freezer mill (SPEX SamplePrep, Metuchen, NJ, USA). The resulting MDM powder was then sieved with a 500 µm filter (Hogentogler & Co. Inc., Columbia, MD, USA) and stored at −80 °C until use. MDM powder was suspended in deionized water to generate 8%, 12%, and 16% by weight MDM slurry, which was homogenized on ice with the PRO 260 homogenizer (PRO Scientific Inc., Oxford, CT, USA) [18,19]. The homogenized MDM slurry was then pipetted into custom 3 mm diameter × 2 mm thick delrin molds, frozen at −80 °C overnight, and re-lyophilized. The resulting MDM scaffolds were then either left non-cross-linked, DHT cross-linked, or genipin cross-linked. Scaffolds that were DHT cross-linked were placed in an oven at 120 °C for 24 h [18,19,23]. Scaffolds that were genipin cross-linked were placed in a solution of phosphate buffered saline (PBS; Gibco, Grand Island, NY, USA) containing 0.05% genipin (Sigma–Aldrich, St. Louis, MO, USA) by weight for 72 h at 37 °C, 5% CO_2_. Genipin cross-linked scaffolds were washed three times with PBS for 5 min, and re-lyophilized [24]. All scaffolds were gas sterilized using ethylene oxide. 

### 4.2. Scanning Electron Microscopy

MDM scaffolds were mounted on a copper covered platform (Ted Pella Inc., Redding, CA, USA) and sputter-coated (Desk V, Denton Vacuum, Moorestown, NJ, USA) with gold at 12 mA for 400 s. Samples were then scanned using the FEI XL30 environmental scanning electron microscope (Hillsboro, OR, USA) at an accelerating voltage of 10 kV and a magnification of 2000×. 

### 4.3. Isolation of Meniscal Cells 

Outer zone meniscal cells were isolated from the medial meniscus of a 6-month-old female porcine knee joint obtained following euthanasia (this study was IACUC exempt as the tissues were obtained as waste following sacrifice for medical school training). The outer zone of the meniscus was minced to ~2 mm × 2 mm pieces and washed in Dulbecco’s modified eagle medium-high glucose (DMEM-HG, Gibco) containing 10% antibiotic–antimycotic (penicillin–streptomycin–fungizone (PSF); Gibco). Meniscus pieces were then enzymatically digested [35,61] with 0.5% pronase (Calbiochem, San Diego, CA, USA) for 1 h followed by overnight digestion with 0.2% collagenase type I (Worthington, Lakewood, NJ, USA) in DMEM-HG containing 10% PSF and 10% fetal bovine serum (FBS; Corning, Corning, NY, USA) at 37 °C. Isolated meniscus cells were then filtered through a 70 µm cell strainer (Corning). Meniscus cells were frozen in liquid nitrogen until further use. 

### 4.4. Primary Meniscus Cell Characterization on MDM Scaffolds

Meniscus cells were plated and expanded in meniscus growth medium consisting of DMEM-HG supplemented with 10% FBS, 1% PSF, 1% 4-(2-hydroxyethyl)-1-piperazine ethanesulfonic acid buffer (HEPES, Invitrogen, Carlsbad, CA, USA), 1% non-essential amino acids (Invitrogen), and 50 μg/mL ascorbic acid 2-phosphate (Sigma–Aldrich) at 37 °C, 5% CO_2_. Passage 1 meniscus cells were suspended at 6.6 × 10^6^ cells/mL and 10 μL of cell suspension was added to each MDM scaffold. Cells were infiltrated into the scaffolds under vacuum for 60 s and this process was repeated on the other side to seed a total of 1.32 × 10^5^ cells per scaffold (*n* ≥ 9 per group). The seeded scaffolds were placed in Costar ultra-low attachment 24-well polystyrene culture plates (Corning) and cultured in 1 mL of meniscus growth medium and were either harvested immediately (day 0) or incubated at 37 °C, 5% CO_2_ for 14 days (*n* ≥ 3 per group), with media changes every 2 days. Biochemical analyses were performed to characterize the response of the meniscus cells to the MDM scaffolds. In order to identify changes in cellular proliferation and ECM composition over time, day 14 biochemical data were normalized to the biochemical data for corresponding day 0 meniscus cell seeded scaffolds. 

### 4.5. Meniscus Repair Model System 

We used an in vitro meniscus repair model system to study the integrative repair of the MDM scaffold with meniscus tissue, as previously described [19]. Briefly, 8 mm diameter explants were harvested from the midline of skeletally mature female porcine medial menisci using biopsy punches (Integra Miltex Inc. York, PA, USA). Explants were trimmed to a thickness of 2 mm with a custom cutting block, leaving the femoral surface of the meniscus tissue explants intact. The explants were then washed with DMEM-HG with 10% PSF for 1 h. To simulate a full-thickness meniscus tear [18,19,33,34,35,36,37,38,39], a 3 mm diameter tissue core was removed from the explant and was either immediately re-inserted in the same orientation (meniscus tissue control), or was replaced with an MDM scaffold. MDM scaffolds tested in these experiments were: 8% non-cross-linked, 8% DHT cross-linked, 8% genipin cross-linked, 12% non-cross-linked, 12% DHT cross-linked, 12% genipin cross-linked, 16% non-cross-linked, 16% DHT cross-linked, and 16% genipin cross-linked (*n* ≥ 5 per group). Samples were cultured in 1 mL meniscus growth medium at 37 °C, 5% CO_2_ for 14 days with media changes every 2 days. 

### 4.6. Fluorescent Imaging of Meniscus Repair Model Constructs 

After 14 days in culture, meniscus repair model constructs were stained for live cells (green) using calcein AM (Invitrogen) and for ECM proteins (red) using Alexa Fluor 633 NHS ester (Invitrogen). Fluorescent images were taken in multiple planes to compile a z-stack (~1mm in depth, starting from the surface of the meniscus repair model explants) on an Olympus IX83 microscope (Olympus, Waltham, MA, USA). The images were then deconvoluted and processed using extended focus imaging (EFI) to compile focused images at each layer of the z-stack into one image [19]. 

### 4.7. Integrative Shear Strength of Repair 

After 14 days in culture, push-out testing was performed to determine the integrative shear strength of repair between the inner MDM core or the inner meniscus tissue core with the outer meniscus tissue ring [18,19,33,34,35,36,37,38,39]. Samples were placed in the center of a custom fixture with a 4 mm diameter hole in the base. The fixture was then placed in a mechanical load frame (ElectroForce 3220 Series III, TA Instruments, New Castle, DE, USA) beneath a 2 mm diameter piston attached to a 250 g load cell (Honeywell, Morris Plains, NJ, USA). A pre-load of 0.25 g was applied and then allowed to equilibrate. Next, the MDM scaffold or tissue core was pushed-out at a constant rate of 0.0833 mm/sec until the piston reached a relative final displacement of 4 mm. Force-displacement curves were generated and the shear strength of repair was defined as the peak force divided by the surface area of the interface. The area of the interface was calculated using the average thickness of each construct measured in Image J (NIH, Bethesda, MD, USA) from images taken by a Genie camera (Teledyne Dalsa, Waterloo, ON, Canada) with a 50 mm lens (Tamron, Cologne, Germany).

### 4.8. Biochemical Analyses 

Following culture and push-out testing, MDM scaffolds were digested in 1 mL of 125 µg/mL papain (Sigma–Aldrich) digestion buffer (0.1M sodium phosphate monobasic monohydrate (Sigma–Aldrich), 5 mM ethylenediaminetetraacetic acid (EDTA; Sigma–Aldrich), 5 mM cysteine hydrochloride (Sigma–Aldrich), pH. 6.5) at 65 °C overnight [18,19,23,62,63]. DNA content was assessed using the Quant-iT Pico-Green^TM^ assay kit (Invitrogen). sGAG content was determined using the dimethylmethylene blue (DMMB) assay with chondroitin sulfate standards isolated from bovine trachea (Sigma–Aldrich) [18,19,62,63,64]. Collagen content was determined by the amount of free OHP produced from alkaline hydrolysis. Colorimetric analysis of OHP was determined following oxidation with chloramine-T (Sigma–Aldrich) and chromophore formation from addition of 4-(Dimethyl-amino)benzaldehyde (Ehrlich’s reagent; Sigma–Aldrich) using trans-4-hydroxyproline (Sigma–Aldrich) as standards [18,19,62,63,65].

### 4.9. Histological Analyses

After 14 days in culture, samples were fixed in 4% paraformaldyhyde (Electron Microscopy Sciences, Hatfield, PA, USA) containing 100 mM sodium cacodylate trihydrate (Electron Microscopy Sciences) pH 7.4 at 4 °C overnight [19,35,36,38,39]. Samples were dehydrated with ethanol (KOPTEC, King of Prussia, PA, USA), xylene (VWR, Radnor, PA, USA) infiltrated, and paraffin (Paraplast, Leica Biosystems, Buffalo Grove, IL, USA) embedded. Samples were sectioned to a thickness of 10 µm and stained with Harris Hematoxylin with glacial acetic acid (Poly Scientific, Bay Shore, NY, USA), 0.02% aqueous fast green solution (Electron Microscopy Sciences), and 0.1% Safranin-O solution (Sigma–Aldrich) to visualize condensed nucleic acid material, collagen, and proteoglycans, respectively [19,38].

### 4.10. Statistical Analyses

Statistical analyses were performed using Statistica 13.3 (TIBCO Software Inc., Chapel Hill, NC, USA). All data were normally distributed. Factorial ANOVA and Fisher LSD post hoc tests were used to detect significant effects (α = 0.05) of MDM percentage and cross-linking on biochemical outcomes and shear strength of repair.

## Abbreviations

ANOVA	analysis of variance
ASC	adipose-derived stem cells
CDM	cartilage-derived matrix
DHT	dehydrothermal
DMEM-HG	Dulbecco’s modified eagle medium-high glucose
DMMB	dimethylmethylene blue
DNA	deoxyribonucleic acid
ECM	extracellular matrix
EDTA	ethylenediaminetetraacetic acid
EFI	extended focused imaging
FBS	fetal bovine serum
GEN	genipin cross-linked
HEPES	4-(2-hydroxyethyl)-1-piperazine ethanesulfonic acid
LSD	least significant difference
MDM	meniscus-derived matrix
hMSC	human mesenchymal stem cells
NON	non-cross-linked
OHP	hydroxyproline
PBS	phosphate buffered saline
PSF	penicillin streptomycin fungizone
SEM	standard error of the mean
sGAG	sulfated glycosaminoglycan
UV	ultraviolet
